# Organoids in skin wound healing

**DOI:** 10.1093/burnst/tkae077

**Published:** 2025-01-03

**Authors:** Zitong Wang, Feng Zhao, Hongxin Lang, Haiyue Ren, Qiqi Zhang, Xing Huang, Cai He, Chengcheng Xu, Chiyu Tan, Jiajie Ma, Shu Duan, Zhe Wang

**Affiliations:** Department of Pathology, Shengjing Hospital of China Medical University, No. 36 Sanhao Street, Shenyang, Liaoning 110004, China; Department of Stem Cells and Regenerative Medicine, Shenyang Key Laboratory of Stem Cell and Regenerative Medicine, China Medical University, No. 77 Puhe Road, Shenyang, Liaoning 110013, China; Department of Stem Cells and Regenerative Medicine, Shenyang Key Laboratory of Stem Cell and Regenerative Medicine, China Medical University, No. 77 Puhe Road, Shenyang, Liaoning 110013, China; Department of Pathology, Wuhan Hospital of Traditional Chinese and Western Medicine (Wuhan No. 1 Hospital), No. 215 Zhongshan Street, Wuhan, Hubei 430022, China; Department of Pathology, Chengdu Third People's Hospital, No. 82 Qinglong Street, Chengdu, Sichuan 610031, China; Department of Anaesthesiology, the First Affiliated Hospital of Xi'an Jiaotong University, No. 277 Yantaxi Road, Xi'an, Shanxi 710061, China; Department of Pathology, Shengjing Hospital of China Medical University, No. 36 Sanhao Street, Shenyang, Liaoning 110004, China; Department of Pathology, Shengjing Hospital of China Medical University, No. 36 Sanhao Street, Shenyang, Liaoning 110004, China; Department of Pathology, Shengjing Hospital of China Medical University, No. 36 Sanhao Street, Shenyang, Liaoning 110004, China; Department of Pathology, Shengjing Hospital of China Medical University, No. 36 Sanhao Street, Shenyang, Liaoning 110004, China; Department of Pathology, Shengjing Hospital of China Medical University, No. 36 Sanhao Street, Shenyang, Liaoning 110004, China; Department of Pathology, Shengjing Hospital of China Medical University, No. 36 Sanhao Street, Shenyang, Liaoning 110004, China

**Keywords:** Skin organoids, Tissue engineering, Pluripotent stem cells, Skin wound healing

## Abstract

Stem cells (SCs) can self-replicate and differentiate into multiple lineages. Organoids, 3D cultures derived from SCs, can replicate the spatial structure and physiological characteristics of organs *in vitro*. Skin organoids can effectively simulate the physiological structure and function of skin tissue, reliably restoring the natural skin ecology in various *in vitro* environments. Skin organoids have been employed extensively in skin development and pathology research, offering valuable insights for drug screening. Moreover, they play crucial roles in skin regeneration and tissue repair. This in-depth review explores the construction and applications of skin organoids in wound healing, with a focus on their construction process, including skin appendage integration, and significant advancements in wound-healing research.

Highlights

- Skin organoids can help to form skin appendages such as sweat glands, sebaceous glands, and hair follicles, which help skin wound to achieve structural and functional reconstruction.
- Microfluidic systems, 3D printing technology, CRISPR-mediated gene editing, and AI-driven high-throughput automation are revolutionizing organoid research, enabling standard organoid molding, precise gene targeting, and efficient drug screening, thus paving the way for skin organoid research.
- Major current issues facing skin organoids include a lack of consistency, challenges with vascularization, the absence of an immune system, and ethical concerns related to clinical translation.

## Background

Stem cells (SCs) are capable of self-renewal and can exhibit multipotency. Under specific conditions, SCs can differentiate into various functional cell types [1] and have the potential to regenerate various tissues and organs. The history of stem cell research dates back to the late 19th century, when scientists began to focus on cells with differentiation and developmental potential. In 1868, the famous German biologist Ernst Haeckel first developed the concept of undifferentiated cells, which was the earliest concept related to SCs. The 1963 publications by Ernest A. McCulloch and James E. Till marked the beginning of modern stem cell research [2]. The use of SCs or their derivatives to repair diseased or damaged tissues overcomes the limitations of conventional clinical treatments and introduces new possibilities for regenerative medicine and the treatment of other human diseases [3,4].

Organoids are 3D cultures derived from SCs that are capable of mimicking the spatial structure and physiological characteristics of organs *in vitro* [5]. Compared with traditional cell cultures, organoids comprise diverse cell types that go beyond making simple physical connections, fostering more complex intercellular communication processes, including interaction, induction, feedback, and collaboration, allowing the organoid to more accurately simulate tissue structure and function [6,7]. Novel advanced biomanufacturing technologies offer the opportunity to design complex cell niches with specific geometries and architectures that influence the spatiotemporal behavior of stem/progenitor cells [8]. With continued advances in organoid technology, researchers have successfully cultivated various organoids, including organoids derived from the brain [9], kidney [10], stomach [11], liver [12], lung [13], mammary gland [12], and pancreas [14]. These organoids serve as invaluable tools for *in vitro* studies of organ development [15], basic research [16], drug discovery [17], and regenerative medicine [18].

The skin, the largest organ in the human body, contains various tissue structures, including the epidermis, dermis, subcutaneous tissue, and appendages. The composition of skin tissue enables it to play a variety of important roles, such as roles in physical protection, temperature regulation, immune defense, secretion, and excretion, as the first barrier through which the body resists infection and injury [19]. Efficient wound repair is crucial for maintaining homeostasis, and research in this field has received increasing attention [20]. The idea of using a skin culture system as an *in vitro* substitute for skin was first proposed by Rheinwatd et al. in 1975 [21]. Those authors pioneered the development of a self-organizing strategy for squamous epithelium generation, which involved serial cocultivation of primary human keratocytes and irradiated 3T3 mouse fibroblasts. This breakthrough paved the way for *in vitro* culture of self-assembled skin tissue.

The emergence and rapid development of skin organoids have brought new opportunities in skin wound healing research, mainly for the following reasons. First, compared with the traditional full-thickness skin model, skin organoids more accurately replicate the *in vivo* development process. These organoids can self-organize and differentiate directionally into different cell types, aligning more closely with the structure and function of native tissues. Furthermore, skin organoids can produce skin appendages, such as hair follicles and sebaceous glands, which are absent in traditional skin models [22], providing a biological environment that closely resembles real skin. Therefore, skin organoids are ideal *in vitro* models for studying the complex process of wound healing. Second, skin organoids have important applications in regenerative medicine. Owing to their ability to simulate the structure and function of real skin, skin organoids are innovative tools for treating skin injuries, burns, and other skin conditions [23,24]. Transplanting skin organoids into the wound site can promote the regeneration and repair of damaged skin [25]. In addition, skin organoids can be used as platforms for drug screening and toxicology studies [26]. By testing drugs or compounds on organoids, their effects on the skin and potential side effects can be predicted. This approach can improve the efficiency of drug development and reduce risks in clinical trials [27].

In recent years, skin organoids constructed *in vitro* have been widely used in skin development research [28,29], skin pathology research [30,31], and drug screening [32,33]. Hong et al. [34] comprehensively summarized the milestones in skin organoid generation and discussed the diverse applications of skin organoids, including their relevance in developmental biology, disease modelling, regenerative medicine, and personalized medicine. This review focuses on the construction and application of skin organoids in wound healing, elaborates on the construction process, and discusses the evolving role of skin organoids in wound healing research.

## Review

### Construction of skin organoids

#### Cell source for the construction of skin organoids

Cells are the fundamental building blocks of an organism, and their proper function is the cornerstone of effective tissue repair and regeneration [35]. Skin organoids are predominantly composed of SCs [36], including adult SCs (ASCs) and pluripotent SCs (PSCs) (Table 1). Many organoids associated with the epidermis, sweat glands, and hair follicles are derived from ASCs [37,38]. PSCs replicate in skin tissue systems *in vitro* through induced differentiation. This approach enables the simulation of the skin and its associated organoids, enhancing the understanding of the complex interactions between different cell types and molecular signaling pathways during development and homeostasis [39,40].

**Table 1:** Comparison of adult stem cells (ACS) and pluripotent stem cell (PSC) derived organoids

	Organoids derived from ASCs	Organoids derived from PSCs
Main source	Organ samples containing SCs	ASC reprogramming, PSC sample bank
Main function	Dynamic maintenance of simulated stem cells	Complete simulation of the development process of the corresponding organs
Differentiation potential	Limited, with a tendency to directly replicate the original tissue phenotype	Pluripotent potential to form more complex structures
Experimental scheme	Few experimental steps, with a short experimental period	Complex and time-consuming cultivation regimen
Organoid characteristics	Consistently reproduces the original tissue type	Heterogeneous cell types similar to the physiological state
Main application	Organoids of surface ectodermal lineages (especially glandular tissue)	Organoids that mimic diseases in human development

##### ASCs

In human skin, various types of ASCs play pivotal roles, including epidermal SCs (EpSCs), dermal SCs (DSCs), and hair follicle SCs (HFSCs). These SCs collaboratively contribute to the development and composition of diverse skin cell lineages, which form the skin. EpSCs are precursors to a wide array of epidermal cells that originate from the embryonic ectoderm and are capable of bidirectional differentiation. Boonekamp et al. [37] established an organoid culture system that enables mouse EpSCs to continually expand and differentiate for an extended period of up to 6 months. DSCs, also known as dermal mesenchymal SCs, undergo differentiation into fibroblasts under specific conditions, and they then stimulate the synthesis and secretion of vital components such as collagen and elastin [41]. Su et al. [42] successfully aggregated DSCs with embryonic stem cells (ESCs) to form hair follicle-like organoids. This innovative approach promoted hair follicle formation both *in vitro* and *in vivo* via WNT pathway activation. HFSCs function as crucial tissue signal centers within the skin, generating rich signal outputs during all stages of adult skin homeostasis. HFSCs play a vital role in regulating the organization and function of skin niches [43]. Chen et al. [44] pioneered the construction of a nanoscale biomimetic extracellular matrix tailored for individual HFSCs. This development facilitated the stable expansion of HFSCs while preserving their essential SC properties. These properties thus markedly influence the outcomes of skin tissue regeneration.

##### PSCs derived from preimplantation embryos (ESCs)

ESCs are pluripotent, self-renewing cells derived from undifferentiated cells originating from preimplantation embryos. Signaling molecules can promote the self-renewal of ESCs and induce the derivation of PSCs. Koehler et al. [45] established 3D mouse ESC cultures to generate a new *in vitro* model of sensory epithelial differentiation in the inner ear to obtain a deeper understanding of inner ear development and disorders. Lee et al. [46] progressively modulated the transforming growth factor β (TGF-β) and fibroblast growth factor (FGF) signaling pathways, co-induced the aggregation of cranial epithelial cells and neural ridge cells into spheres, constructed an organoid culture system capable of generating complex skin directly from human ESCs and successfully used it for skin reconstruction *in vivo*. Furthermore, a 3D mouse ESC culture was developed to spontaneously produce new hair follicles that mimic their normal counterparts [29].

##### Induced PSCs

Induced PSCs (iPSCs) are a category of PSCs with the capacity for infinite self-renewal and proliferation and can differentiate into mature cells of the ectoderm, mesoderm, and endoderm [47]. iPSCs can be generated from somatic cells, including fibroblasts, keratinocytes, and blood cells, through a process known as reprogramming [48]. This approach overcomes the immunological concerns associated with ESCs. Furthermore, iPSCs can differentiate into diverse cell types, including keratinocytes and fibroblasts [49], providing a rich source of cellular components for constructing skin organoids. Yang et al. [50] enriched keratinocytes in culture dishes and transfected them with lentivirus encoding transcription factors to obtain epidermal cells and generate iPSCs. Kim et al. [51] reported that iPSCs derived from human cord blood mononuclear cells exhibited high pluripotency, normal karyotypes, and the ability to differentiate into all three blastoderm layers. Keratinocytes and fibroblasts derived from these iPSCs presented characteristics similar to those of primary cell lines. Sahet et al. [52] employed a coculture approach in which iPSC-derived fibroblasts and keratinocytes were utilized to produce 3D skin equivalents. Itoh et al. [53] generated iPSCs from fibroblasts and directed their differentiation into keratinocytes, resulting in the production of functional 3D skin equivalents. Abbas et al. [54] generated skin organoids from human iPSCs derived from human skin fibroblasts or placental CD34+ cells, produced complex skin organoids with skin layers and pigmented hair follicles, and successfully developed sebaceous glands, tactile receptive Merkel cells, and secretory sweat glands.

#### Scaffolds for skin organoids

Organoid construction often requires the regulation of physical signals. Hydrogels mimic the *in vivo* environment through their unique physical, chemical, and biological properties, providing essential signaling support for skin cell growth and differentiation [55]. In terms of physical properties, the 3D cross-linked polymer network of the hydrogel provides cells with a 3D scaffold similar to the structure of the extracellular matrix *in vivo*. This 3D environment facilitates the appropriate arrangement and interaction of cells in space to mimic complex tissue structures *in vivo*. Second, the mechanical properties of hydrogels (such as hardness and elasticity) can be adjusted by changing their degree of cross-linking, polymer concentration, and other parameters. This flexibility allows researchers to precisely control the mechanical environment according to different organoid needs, mimic the mechanical properties of different tissues in the body, and provide a growth space for cells that is similar to that *in vivo* [56]. In terms of biochemical characteristics, hydrogels can promote interactions between cells and biochemical signaling; affect cell morphology, growth rate, and differentiation direction; prevent excessive proliferation and migration; maintain structural stability and organoid function; and facilitate organoid reproduction *in vitro*, thereby increasing the complexity and functionality of the tissue [57].

When choosing hydrogels, factors such as chemical composition, cross-linking degree, elastic modulus and other factors should be considered [58]. During the preparation process, the properties of a hydrogel can be regulated by changing its type, concentration, and cross-linking conditions [59]. Hydrogels with different biochemical properties and physical properties can simulate different *in vivo* environments, thereby regulating the growth and differentiation of cells and promoting the formation and maturation of skin organoids [60]. Hydrogels can also serve as carriers for drugs or growth factors to promote cell growth and differentiation and accelerate skin organoid formation. One team designed a microfluidic device to produce an asymmetric gradient of differentiation factors in a spindle hydrogel to improve the spatial organization of dermal and epidermal cells, promoting keratinocyte differentiation and hair follicle formation in skin organoids [61].

### Types of skin organoids

**Figure 1:**
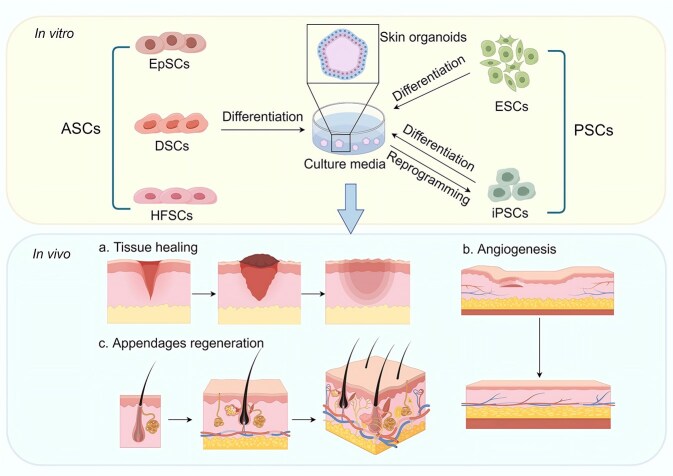
***In vitro* composition and *in vivo* functions of skin organoids.**  *In vitro*, ASCs and PSCs retain the differentiation potential of stem cells in skin organoids and provide rich and sufficient cell components for constructing skin organoids. iPSCs can be generated from somatic cells through reprogramming and can also differentiate into various types of cells. When transplanted *in vivo*, skin organoids promote skin tissue healing, neurovascular repair, and the regeneration of appendages. *ASC* adult stem cell, *PSC* pluripotent stem cell, *iPSC* induced pluripotent stem cell

The epidermal layer of the skin produces hair and glands. This layer is primarily composed of keratinocytes, which aid in thermoregulation and barrier formation. The dermis, underneath the epidermis, houses an array of structures, including blood vessels and nerves. Dermal fibroblasts within this layer are prolific producers of extracellular matrix components, such as collagen and elastic fibers. These elements provide skin with its well-known elasticity and facilitate the initiation and circulation of hair follicles [62]. The subcutaneous fat lying beneath the dermis serves as an energy reservoir within subcutaneous tissues. Moreover, the skin contains sweat glands, sebaceous glands, and hair follicles derived from the epidermal and dermal layers. A variety of epidermal organoids, as well as organoids containing skin appendages, such as hair follicle and sebaceous and sweat gland organoids, have been constructed for scientific research and clinical treatment (Figure 1).

#### Epidermal organoids

Boonekamp et al. [37] cultured epidermal keratinocytes extracted from the dorsal skin surface of mice to obtain mouse epidermal organoids for long-term expansion and differentiation, contributing to the study of epidermal homeostasis *in vitro*. Xie et al. [63] constructed mouse primary epidermal organoids, which presented stratified histological and morphological features resembling those of the epidermis. These organoids closely simulate their native tissues at the transcriptomic and proteomic levels, which is valuable for skin infection modelling and drug screening. Wiener et al. [64] seeded cells obtained from microdissected interfollicular epidermis within a basement membrane extract to generate epidermal organoids. This method shows promise as an *in vitro* model for exploring epidermal structure, function, and dysfunction. Wang et al. [65] harnessed freshly isolated human protodermal cells to establish 3D cultures of human primary epidermal organoids, which served as an effective model for studying dermatophyton infections. In their most recent study, Kwak et al. [66] developed multipotent stem cell-derived epidermal organoids, which produce efficient extracellular vesicles for skin regeneration and contribute to target cell proliferation, migration, and angiogenesis, showing promise as therapeutic tools for wound healing *in vivo*.

#### Hair follicle organoids

Hair follicles are sac-like structures located within the dermis and subcutaneous tissue that are responsible for hair growth. The hair follicle has two main parts: the upper part, which includes the infundibulum and isthmus, and the lower part, which includes the bulb and suprabulbar region. Gupta et al. [67] constructed *in vitro* 3D organoid models encapsulated in sericin hydrogels containing human hair dermal papilla cells, hair follicle keratinocytes, and SCs. These models exhibited structural features akin to those of natural hair follicles, mimicking cell–cell interactions and a hypoxic environment. Ramovs et al. [68] successfully produced hair-skin organoids from two human iPSC lines and thoroughly characterized epidermal junctions via immunofluorescence and transmission electron microscopy. Weber et al. [69] combined neonatal foreskin keratinocytes with scalp dermal cells and successfully established hair peg-like structures *in vitro* that expressed appropriate epidermal and dermal markers. This method serves as a platform for optimizing the engineering of human hair follicles for transplantation. Veraitch et al. [70] reported that ectodermal precursor cells derived from human iPSCs were able to communicate with hair-induced dermal cells, ultimately promoting hair follicle formation. Marinho et al. [71] developed a technique to construct hair follicle organoids *in vitro* by combining multiple cell types. When these organoids were transplanted into the skin, structural fusion and hair bud generation occurred. Kim et al. [72] developed solar UV-exposed skin organoids derived from human iPSCs, which effectively recapitulated several symptoms of photodamage, including skin barrier disruption, extracellular matrix degradation and the inflammatory response.

#### Sebaceous gland organoids

The sebaceous gland is a notable skin appendage that is widely distributed across the body, except for the hands and feet. Secreted sebum combines with sweat to create a lipid membrane that is crucial for skin protection. Feldman et al. [73] used Blimp1+ cells isolated from adult mice to replicate sebaceous gland lineage expression and homeostasis dynamics *in vitro*. The authors successfully established sebaceous gland organoids, which serve as valuable tools for drug screening and investigations into sebaceous gland homeostasis, function, and pathology. Wang et al. [74] demonstrated that functional hair follicles and sebaceous glands could be reconstituted by transplanting a combination of culture-expanded ESCs and skin-derived progenitors from mice and adult humans.

**Figure 2:**
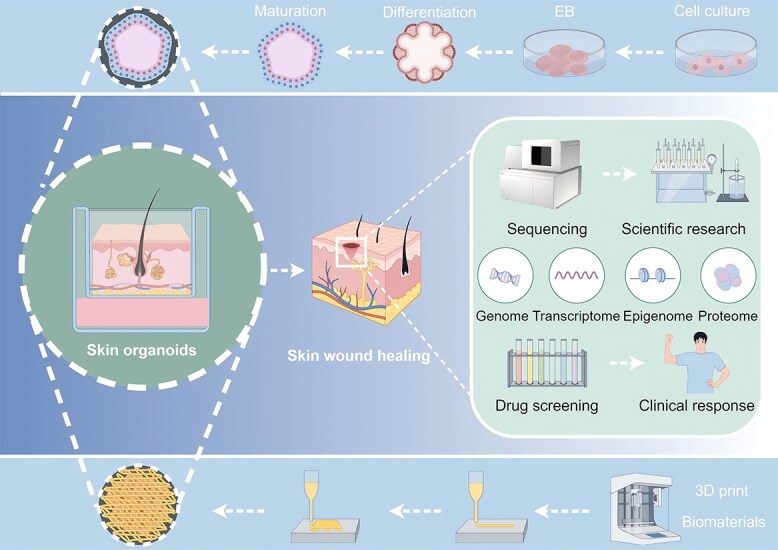
**Future directions for skin organoids in skin wound healing.** Patient-specific iPSCs manufactured from skin biopsies and 3D-printed organoids promote skin wound healing. In the future, the combination of skin organoids and sequencing technologies, including genome sequencing, transcriptome sequencing, epigenetic sequencing, and proteome sequencing, will provide new insights for scientific research on skin wound healing. Drug screening can also be performed in skin organoids to predict the clinical efficacy of drugs for burn and trauma patients. *iPSC* induced pluripotent stem cell, *3D* three-dimensional

#### Sweat gland organoids

Sweat glands secrete sweat, excrete waste, maintain body temperature, and originate from epidermal progenitor cells, similar to other skin components. The regenerative ability of sweat glands after full-thickness injury is limited, and the repair and regeneration of the sweat gland structure and function after severe burns remain major challenges in clinical treatment [75,76]. Diao et al. [38] embedded sweat gland epithelial cells in matrix glue in the dermis of the paw pads of adult mice, maintaining SC characteristics to enable differentiation into sweat glands or epidermal cells, effectively integrating sweat glands into tissues, and successfully establishing mouse sweat gland organoids. Sun et al. [77] reprogrammed human epidermal keratinocytes to differentiate into a lineage of sweat gland cells capable of sweat gland regeneration. Yuan et al. [78] generated replicable spheres of sweat gland cells from adipose mesenchymal SCs and formed blood vessels with dermal microvascular endothelial cells, successfully simulated the morphogenesis of vascularized glands *in vitro*, and reported the precise anatomical relationships and interactions between sweat gland cells and the surrounding vascular niche.

### Application of skin organoids in wound healing

Skin damage from surgery, trauma, or burns has considerable physical and psychological effects on patients [79]. Skin organoids offer a promising avenue for overcoming the challenges posed by hard-to-heal wounds and the permanent loss of skin appendages. Currently, a variety of biological structures and clinical strategies are being employed to harness the potential of skin organoids to address clinical issues related to wound healing (Figure 2).

#### Traditional skin organoid transplantation

At present, the organoids used in skin wound healing are derived predominantly from iPSCs. Takagi et al. [80] generated 3D human skin organoids from iPSCs, incorporating accessory organs such as hair follicles and sebaceous glands that exhibited full functionality after transplantation into nude mice, effectively integrating with surrounding host tissues, including the epidermis, arrector pili muscles, and nerve fibers. Ma et al. [81] established epithelial and mesenchymal organoid models derived from human induced pluripotent SCs, which enhanced epidermal stem cell activity, promoted sweat gland and blood vessel regeneration and provided new therapeutic options for skin lesions and functional defects. Lee et al. [46] developed complex skin organoids from human PSCs, which comprised a layered epidermis, a fat-enriched dermis, and pigmented hair follicles complete with sebaceous glands. When these skin organoids were transplanted into nude mice, the epidermal layer was oriented to create hair follicles, which resulted in the formation of planar hair-bearing skin. This innovation holds promise for skin reconstruction in patients with burns or trauma. Ebner–Peking et al. [25] differentiated human tissue-derived iPSCs into endothelial cells (ECs), fibroblasts, and keratinocytes to generate a cell suspension, which promoted full-thickness wound healing in mice *in vivo*. Diao et al. [38] embedded epithelial cells derived from sweat glands in the dermis of the paw pads of adult mice using Matrigel, forming sweat gland organoids that retained SC characteristics. These organoids had the capacity to differentiate into sweat gland cells or epidermal cells, and *in vivo* experiments confirmed that such organoids could enhance skin wound healing and sweat gland regeneration. Thai et al. [82] co-cultured ECs and mesenchymal SCs (MSCs) to form EC-MSC spheres encapsulated within hydrogels that promoted wound healing in an *in vitro* full-thickness skin burn model.

Organoids constructed from reprogrammed skin cells can also be used in wound healing research. Sun et al. [77] employed reprogrammed human epidermal keratinocytes to construct regenerative sweat gland organoids. These organoids were subsequently transplanted into a skin injury mouse model, resulting in the successful development of fully functional sweat glands. For refractory diabetic wounds, Choudhury et al. [83] creatively transdifferentiated chemokine receptor allogenic mesenchymal SCs overexpressing Cxcr2 into keratinocyte-like cells in 2D and 3D cell culture. After these organoids were transplanted into a diabetic mouse wound healing model, epithelialization of the epidermal layer and endothelialization of the dermal layer significantly increased, notably increasing the wound closure rate.

#### Skin organoid transplantation combined with 3D printing technology

To date, numerous studies have employed skin cells as seed cells for 3D printing to form skin organoids, which have been applied in wound healing research. Cubo et al. [84] utilized keratinocytes and fibroblasts as seed cells for 3D printing skin tissue. By using histological and immunohistochemical analyses both *in vitro* and *in vivo*, the authors demonstrated that the printed skin closely resembled normal human skin in terms of structure and function. This printed skin could mimic various physiological properties of human skin, holding promise for future applications in scientific and clinical research on skin wound healing. In another study, six primary human skin cell types were used to bioprint a three-layer skin construct comprising the epidermis, dermis, and hypodermis. The bioprinted skin organoids were transplanted into full-thickness skin injury models of mice and pigs, where they achieved full integration and regenerated skin, promoted skin neovascularization and extracellular matrix remodeling, and accelerated wound healing [85].

Moreover, 3D printing can control the spatial arrangement of cells in skin organoids to facilitate skin reconstruction. Pappalardo et al. [86] employed 3D-printed skin tissue for skin reconstruction, successfully replicating biophysical interactions and cellular/extracellular tissue dynamics in human skin. Compared with traditional hydrogel skin organoid transplantation, this approach results in superior mechanical resistance and angiogenesis potential. It can effectively replace full-thickness wounds with minimal sutures and reduce surgery duration. Abaci et al. [87] harnessed 3D printing technology to control the spatial arrangement of cells within a bioengineered human skin structure. The authors initiated dermal cell formation by controlling the autoaggregation of globules within a physiologically relevant extracellular matrix, which facilitated epidermal–mesenchymal interactions. This innovative approach led to hair follicle formation *in vitro*, offering new possibilities for research into hair follicle regeneration following scalp trauma.

In addition, 3D printing of organoids via laser-assisted bioprinting (LaBP) technology and digital light processing (DLP) technology has been applied in skin wound healing research. For example, LaBP technology was employed to 3D print fibroblasts and keratinocytes onto a stable matrix, forming a fully cellular skin substitute. In vivo experiments confirmed that this skin substitute, when grafted onto full-thickness skin wounds, accelerated the formation of new blood vessels and promoted the healing of dorsal skin wounds in mice [88]. DLP-based 3D printing technology can enable the precise positioning of clusters of human skin fibroblasts and human umbilical vein ECs with high cell viability. This technology facilitates the generation of functional living skin (FLS) that can be easily implanted into wound sites to promote neovascularization and skin regeneration [89]. FLS mimics the physiological structure of natural skin and displays robust mechanical and bioadhesive properties.

#### Skin organoid transplantation combined with biomaterials

The combination of skin organoids with novel biomaterials also provides new approaches for skin wound healing. Huang et al. [90] and Yao et al. [91] used alginate/gelatine hydrogels as bioinks for 3D printing of the extracellular matrix to simulate the regenerative microenvironment, spatially integrate a variety of biophysical and biochemical cues for cell regulation, promote the transformation of epithelial progenitor cells and mesenchymal SCs into functional sweat glands, and promote sweat gland tissue recovery in mice. Kang et al. [92] constructed a multilayer composite scaffold with epidermal and dermal structures using gelatine/alginate gel. After the scaffold was transplanted into full-thickness wounds in nude mice, it exhibited good cytocompatibility, increased the proliferation ability of dermal papillary cells (DPCs), promoted the formation of self-aggregating DPC spheres, and initiated cuticle–mesenchymal interactions, promoting the formation of hair follicles. Two types of polymer mesh were physically strengthened and integrated into a type I collagen hydrogel to generate a novel dermoepidermal skin substitute; when this platform was transplanted into rats, it uniformly developed into a well-layered epidermis and formed a well-vascularized dermal component [93]. Zhao et al. [94] prepared an alginate–gelatine composite hydrogel bioink with platelet-rich plasma (PRP) integration. The inclusion of PRP not only improved the extracellular matrix synthesis, but also regulated the vascularization of vascular Ecs and macrophage polarization in a paracrine manner. This approach accelerated high-quality wound healing in a rat dorsal full-thickness wound model, demonstrating the remarkable feasibility of 3D bioprinting combined with a PRP-functionalized bioink for expediting wound healing. Bacakova et al. [95] developed a bilayer skin structure composed of collagen hydrogels enhanced with a nanofiber poly(L-lactic acid) membrane of preseeded fibroblasts, which could promote fibroblast adhesion, proliferation and migration to collagen hydrogels. In addition, this construct induced keratinocytes to form the basal and upper layers of cells with high mitotic activity, which could be used for cases of full-thickness skin damage. Guo et al. [96] cross-linked recombinant human collagen (rHC) and transglutaminase to prepare rHC hydrogels and embedded fibroblasts to develop a new tissue-engineered skin equivalent with good biocompatibility, which can promote fibroblast migration and the secretion of a variety of growth factors. This construct has been shown to significantly promote skin wound repair in a full-thickness skin defect mouse model.

#### Skin organoid transplantation combined with growth factors

The TGF-β and FGF signaling pathways are the main regulators of skin cell induction, fate determination, migration, and differentiation [97]. The addition of basic FGF-2 stents can promote neovascularization in the dermis, thus further enhancing the repair of full-thickness skin defects [98]. Lee et al. [46] reported that complex skin organoids derived from human pluripotent SCs gradually regulate TGF-β and FGF and that transplantation of these skin organoids into the hairy skin of nude mice results in the formation of smooth hairy skin.

Currently, WNER (Wnt-3a, Noggin, EGF and R-Spondins) is the classic cytokine culture protocol used in organoid culture because fluctuations in the levels of these four factors are relevant for almost all organoid culture experiments. Other studies have shown that the addition of other small molecules, such as CHIR99021 and valproic acid, to ENR (epidermal cell growth factor (EGF), Noggin, and R-Spondins) can induce specific differentiation of SCs [99]. The study of Kageyama et al. [100] confirmed that adding oxytocin to hair follicle organoids upregulated expression of the growth factor VEGF-A and promoted the growth of hair- and nail-like buds. EGF, FGF, TGF-β, and other growth factors have been applied in wound treatment and in the culture of skin organoids. The use of appropriate concentrations of these growth factors is expected to promote cell proliferation and differentiation, improve wound healing speed and quality, and reduce scar formation and infection risk.

#### Discussion and prospects

The currently established skin organoids follow a natural developmental pathway involving the directional differentiation of SCs to replicate the structure and function of *in vivo* tissue. These organoids play an increasingly vital role in research on skin development, skin disease pathology, and drug screening.

Wound healing approaches using skin organoids have the following advantages. First, skin organoids can be generated *in vitro* to simulate the wound healing process, accelerating drug screening and therapy development. Second, skin organoids derived from patients themselves have a high degree of personalization, which enables them to better simulate the wound environment of patients and improve the precision and effectiveness of treatment. Third, performing drug screening and treatment development *in vitro* is safe and reduces risk and uncertainty in clinical trials to improve treatment safety.

As *in vitro* models, skin organoids have the ability to simulate skin structure and function, providing an important platform for in-depth studies of skin development, disease mechanisms and drug screening. However, their generation process is relatively complex and time-consuming, with concerns related to standardization and diversification, which are major challenges in current research and related applications. Nonetheless, with continuous advances in technology, we can expect to overcome these limitations in the future to better leverage the potential of skin organoids in wound healing and other fields.

One feature worth noting is that organoid cultures lack consistency, making standardized production difficult to achieve. The inconsistency of organoid cultures can be attributed to challenges related to strictly controlling the source, state, and culture conditions of cells [101], which hinders their clinical applicability. Therefore, in the preliminary research phase, extensive clinical, genetic, and morphological data must be integrated to construct a more stable and clinically suitable organoid model [102,103]. Researchers and enterprises should strengthen the collaboration between medicine and industry and integrate and innovate existing technology [104] by developing new bioactive materials and establishing standardized processes, standards, and quality control methods. This would enhance the stability, biocompatibility, and degradability of skin organoids and reduce the risks associated with clinical use, making these organoids more widely applicable [105].

In addition, angiogenesis plays a crucial role in wound healing [106], and vascularized organoids can recreate the interaction between the parenchyma and blood vessels, restoring a realistic skin environment [8,107]. The incorporation of angiogenesis-related factors into skin organoids via advanced biotechnology can regulate biological signal transmission and accelerate blood vessel formation [108]. Furthermore, microfluidic systems simulating blood vessels have been employed to increase blood vessel formation and perfusion in skin organoids [109,110]. In a recent study, one team developed a microfluidic platform that connects the vascular network to organoids and improves the growth and maturation of 3D vascular organoids produced with human-induced pluripotent SCs [111]. Wang et al. [112] constructed a 3D vascular fluidized organ chip based on open microfluidic control, providing a method for realizing *in vitro* construction of vascularized organoid models. This method can be further applied to combine organoids with vascularized organ chips to culture vascularized organoids and resolve the challenge of vascularization in organoid culture.

Another important challenge related to skin organoids is the lack of an immune system. The skin is an important part of the body’s immune defense system and has the ability to recognize and resist invasion by foreign pathogens. However, the currently established skin organoids lack the immune components required to adequately recapitulate human skin biology and disease complexity, limiting their ability to simulate real skin function fully. Currently, Bouffi et al. [113] have deciphered human gut–immune crosstalk during development and developed organoids containing immune cells by transplanting intestinal organoids under the kidney envelope of mice with a humanized immune system. Another research team has jointly developed functional macrophages in human colonic organoids derived from multipotent SCs. These macrophages regulate cytokine secretion in response to proinflammatory and anti-inflammatory signals, perform phagocytosis, and respond to pathogenic bacteria [114]. These developments provide completely new ideas for the construction of skin organoids that simulate the immune response in the skin.

Finally, the clinical translation of organoids raises considerable ethical concerns. Compared with some cell types that have been widely used in clinical practice (such as red blood cells and platelets), there are more ethical concerns about the safety, efficacy and long-term impact of skin organoids in clinical applications because of their unique regenerative potential and undifferentiated state. Moreover, the utilization of organoids derived from patient-specific ASCs or iPSCs for drug testing can be a valuable way to tailor treatments to individual patients. However, such patient-specific trials are costly and offer limited benefits, preventing them from passing cost–benefit evaluations during ethical review. From a technical safety standpoint, organoid transplantation involves invasive surgery, and the uncontrolled development of SCs may pose substantial risks, making predictions based on animal models challenging. The International Society for Stem Cell Research has issued guidelines for human SC research and clinical translation [115]. It is crucial to carefully study and assess potential ethical issues in research on and applications using organoids; improve the corresponding laws, regulations, and scientific research ethics guidelines; and standardize the research and application of these organoids. This proactive approach will contribute to the responsible and ethical development of the SC field.

**Figure 3:**
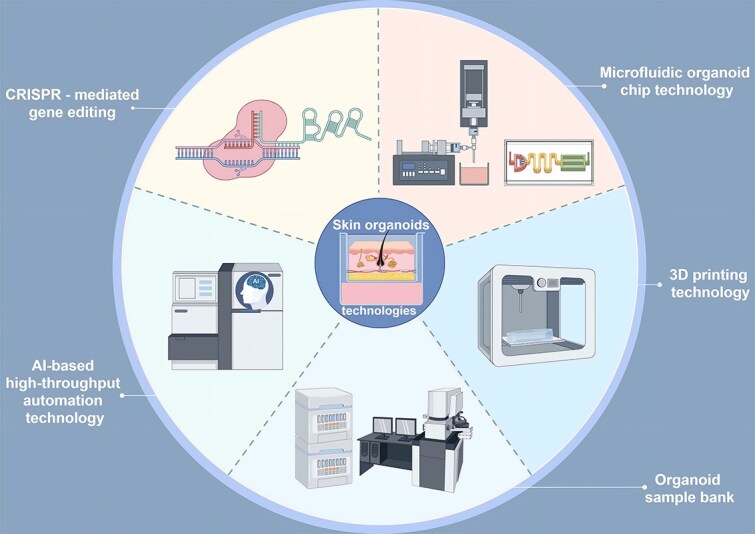
**Key organoid-related technologies for promoting skin wound healing in the future.** The key technologies for promoting skin wound healing in the future include CRISPR-mediated gene-editing technology, microfluidic organoid chip technology, 3D printing technology, high-throughput automation technology based on AI, and organoid sample bank establishment. These key technologies hold great promise for skin wound healing and improving patient outcomes in the future. *3D* three-dimensional, *AI* artificial intelligence

The future focus of research on skin organoids mainly includes CRISPR-mediated gene-editing technology, microfluidic organoid chip technology, 3D printing technology, high-throughput automation technology based on artificial intelligence (AI), and organoid sample bank establishment (Figure 3).

Targeting key endogenous genes via CRISPR technology and increasing their expression may contribute to skin wound repair. In 2020, Artegiani et al. [116] achieved fast and efficient knock-in of human organoids via the nonhomology-dependent CRISPR-Cas9 technology CRISPR-HOT (CRISPR-Cas9-mediated homology-independent organoid transgenesis), providing a vital platform for endogenous knock-in in human organoids. Dekkers et al [117] modelled breast cancer via CRISPR-Cas9-mediated engineering of human breast organoids. Michels et al [118] developed a platform for pooled CRISPR–Cas9 screening in human colon organoids, which was helpful for screening for tumor suppressors both *in vitro* and *in vivo*. Mircetic et al [119] pioneered the use of negative selection-based CRISPR screening for patient-derived organoids and identified a cohort of patients who may benefit from gene-targeting therapy. In the field of skin organoid models, Dabelsteen et al [120] used CRISPR-Cas9 gene targeting to generate a library of 3D organotypic skin tissues that selectively differ in their capacity to produce glycan structures on the main types of N- and O-linked glycoproteins and glycolipids.

Engineering solutions based on microfluidic and 3D printing technology can resolve issues related to the difficulty of molding organoids, the short modelling and molding time, and small sample sizes, thereby enabling the transition of skin organoids from research and development to commercial application as standardized clinical tools. Organ chips based on microfluidic technology can replicate and regulate multiple microenvironments within microfluidic devices. They offer advantages in terms of the controllability and standardization of modelling, enabling the construction of more complex skin models [121]. 3D printing technology can not only support the long-term growth of cells under laboratory conditions but also simulate the mechanical properties of real organs, providing strong technical support for the *in vitro* culture of skin organoids.

AI high-throughput automation can be applied to sample quality control and standardization of the culture and use process, improving the success rate, optimizing and reducing the time associated with manual procedures, and facilitating clinical application. First, image analysis technology combined with deep learning can more accurately capture the microstructure and changes of organoids, improve the ability to identify changes in their morphology and growth, provide accurate data support for experiments, and reduce time and costs [122]. Second, omics data from organoids provide new tools for the resolution of cell development and disease mechanisms [123]. In drug screening, a key application of organoids, AI enables real-time monitoring of drug activity, which enhances screening accuracy and efficiency [124]. In the future, AI is expected to play a greater role in the study of skin organoids, accelerating their clinical translation and the development of precision treatment.

The establishment of biobanks is conducive to the cultivation and maintenance of organoid models, collaborative scientific research among researchers, and the transformation of scientific research results into market applications. By establishing a large-scale library of organoid samples, many experimental materials can be generated to provide accurate and reliable data support for experiments [125]. In addition, diverse samples can simulate the physiological state of the skin in different populations and under different healing conditions, providing a more comprehensive reference for drug development and wound treatment [126].

## Conclusions

Skin organoids are emerging as promising models and treatment strategies for skin wound healing, offering novel avenues for scientific research and clinical interventions. With ongoing advances in technologies, such as 3D printing, culture systems for skin organoids are continually maturing, evolving from simple *in vitro* cultures to complex systems encompassing the epidermis, dermis, and appendages. These systems can facilitate skin cell regeneration and help establish a microenvironment conducive to skin wound healing. However, skin organoid technology currently has several limitations, and related research has yet to comprehensively meet clinical use requirements. With the continuous refinement of skin organoid culture systems, translation from basic research to clinical applications can be expected soon. This approach will enable functional repair and regeneration of wounded skin, ultimately benefiting a substantial number of patients with skin burns and trauma.

## Data Availability

Not applicable.
